# Xanthine oxidase levels and immune dysregulation are independently associated with anemia in *Plasmodium falciparum* malaria

**DOI:** 10.1038/s41598-023-41764-4

**Published:** 2023-09-07

**Authors:** Marilyn Vasquez, Margaux Sica, Ruth Namazzi, Robert O. Opoka, Julian Sherman, Dibyadyuti Datta, Miquel Duran-Frigola, John M. Ssenkusu, Chandy C. John, Andrea L. Conroy, Ana Rodriguez

**Affiliations:** 1 New York University School of Medicine, 430E 29th St, New York, NY 10016 USA; 2 Department of Paediatrics, Makerere University College of Health Sciences, Kampala, Uganda; 3 Global Health Uganda, Kampala, Uganda; 4 Ryan White Center for Pediatric Infectious Disease and Global Health, Indiana University School of Medicine, Indianapolis, IN 46202 USA; 5 Center for Global Health, Indiana University, Indianapolis, IN 46202 USA; 6 Ersilia Open Source Initiative, 28 Belgrave Road, Cambridge, CB1 3DE UK; 7 Department of Epidemiology and Biostatistics, Makerere University School of Public Health, Kampala, Uganda

**Keywords:** Parasite host response, Malaria

## Abstract

Severe anemia is an important contributor to mortality in children with severe malaria. Anemia in malaria is a multi-factorial complication, since dyserythropoiesis, hemolysis and phagocytic clearance of uninfected red blood cells (RBCs) can contribute to this syndrome. High levels of oxidative stress and immune dysregulation have been proposed to contribute to severe malarial anemia, facilitating the clearance of uninfected RBCs. In a cohort of 552 Ugandan children with severe malaria, we measured the levels of xanthine oxidase (XO), an oxidative enzyme that is elevated in the plasma of malaria patients. The levels of XO in children with severe anemia were significantly higher compared to children with severe malaria not suffering from severe anemia. Levels of XO were inversely associated with RBC hemoglobin (ρ =  − 0.25, *p* < 0.0001), indicating a relation between this enzyme and severe anemia. When compared with the levels of immune complexes and of autoimmune antibodies to phosphatidylserine, factors previously associated with severe anemia in malaria patients, we observed that XO is not associated with them, suggesting that XO is associated with severe anemia through an independent mechanism. XO was associated with prostration, acidosis, jaundice, respiratory distress, and kidney injury, which may reflect a broader relation of this enzyme with severe malaria pathology. Since inhibitors of XO are inexpensive and well-tolerated drugs already approved for use in humans, the validation of XO as a contributor to severe malarial anemia and other malaria complications may open new possibilities for much needed adjunctive therapy in malaria.

## Introduction

Malaria is a major cause of anemia in the areas of the world where this disease is endemic^1^. Severe anemia is a common complication of malaria, which occurs most frequently in children under the age of five and is a major contributor of death by malaria^1^. Although mortality associated with severe anemia in children with malaria is low with appropriate transfusion, unreliable blood availability in hospitals frequently results in avoidable deaths^2^. Additionally, children with severe malarial anemia (SMA) are at risk of repeated malarial episodes and increased post-discharge mortality^3^.

Malaria-induced anemia involves both dyserythropoiesis and increased loss of infected and uninfected red blood cells (RBCs)^1,4^. While *Plasmodium* infection of RBCs can lead to destruction and clearance of these cells, the majority of RBCs lost during *Plasmodium* infection are uninfected RBCs. For each RBC lysed directly due to *Plasmodium* infection, about eight uninfected erythrocytes are lost in *P. falciparum*^5,6^ infections. Although the mechanisms underlying the loss of uninfected RBCs during malaria are not completely understood, two mechanisms have been proposed: (1) increased RBCs rigidity from oxidative stress leads to subsequent splenic clearance of these cells^7,8^, and (2) immune dysregulation resulting in binding of antibodies and immune complexes (IC) to uninfected RBCs^9,10^ together with complement dysfunction^11,12^ would contribute to increased uptake by macrophages.

Oxidative stress is elevated during malaria and has been associated with different complications^13^, including anemia^7,14^, but also respiratory distress and acute kidney injury, as well as mortality after 6 months^15,16^. Here we have determined the levels of xanthine oxidase (XO), an oxidative enzyme not found in RBC^17^ that is upregulated during malaria^18^, in a cohort of Ugandan children with severe malaria. We have observed a highly significant correlation between the levels of this enzyme and severe anemia in these patients. We also measured the levels of other immune-related parameters previously described to correlate with SMA: anti-phosphatidylserine (anti-PS) antibodies^19–23^, anti-DNA antibodies^21,23^ and immune complexes (IC)^10^, finding that they are only weakly associated with the levels of XO. Taken together, these results indicate that XO is associated with SMA independently of autoimmune-related mechanisms. A significant association of XO levels and other common complications of severe malaria suggests a possible broad relation of XO with malaria-induced pathology.

## Methods

### Study population

Ethics and consent to participate: Written informed consent was obtained from parents or guardians of study participants. Ethical approval was granted by the Institutional Review Boards for human studies at Makerere University School of Medicine and Indiana University School of Medicine. All methods were performed in accordance with the relevant guidelines and regulations.

As described before^24^, between 2014 and 2017, 600 children with severe malaria were enrolled in the study designed to assess cognition in children < 4 years of age at 12 months’ follow-up^25^. Children were eligible if they: (1) were between 6 months and 4 years of age; (2) had diagnostic evidence of malaria with either a positive rapid diagnostic test for *P. falciparum* histidine-rich protein-2 (HRP2) or direct visualization of parasites by Giemsa microscopy; (3) required hospitalization; and (4) had 1 or more of the following severe malaria criteria: coma (Blantyre coma score < 3), respiratory distress (deep acidotic breathing or lower chest wall retractions), multiple seizures (≥ 2 generalized seizures in 24 h or a seizure > 30 min in duration), severe anemia (hemoglobin ≤ 5 g/dL), or prostration (≥ 1 year, unable to sit unsupported or stand; < 1 year, unable to breastfeed). Children were recruited at 2 referral hospitals in Central and Eastern Uganda: Mulago National Referral Hospital in Kampala and Jinja Regional Referral Hospital in Jinja. Exclusion criteria included the following: known chronic illness requiring medical care, history of coma, head trauma, known developmental delay, cerebral palsy, or prior hospitalization for malnutrition. Delayed exclusion criteria included an elevated cerebrospinal fluid white blood cell count in a child with coma. Children with severe malaria returned for an interim health assessment and blood draw at one-month follow-up.

For controls, 120 community children from the nuclear family, extended family, or household area of the children with severe malaria were enrolled. Additional exclusion criteria for the community children included an illness requiring medical care within the previous four weeks, a major medical or neurologic abnormality at screening physical examination, or active illness. Samples from 98 children in this group were used for this study. Samples from 11 children in this group presented positive levels of HRP2 (> 100 ng/mL), of which five were confirmed by Giemsa microscopy.

Other clinical complication definitions: Acute kidney injury (1.5-fold increase in creatinine over estimated baseline or a 0.3-mg/dL change in creatinine within 48 h)^26^; Acidosis: base deficit of > 8 mmol/L or, if unavailable, a plasma bicarbonate of < 15 mmol/L or venous lactate of > 5 mmol/L^27^.

At enrollment, study participants had serum collected for long-term storage at − 80 °C. All children had a complete blood count, blood smear to quantify malaria, stool testing for helminth infection, and were tested for HIV. At one month follow up the children with severe malaria returned for a health assessment and had serum collected and stored at − 80 °C.

### Measurement of XO activity

XO activity in plasma samples from this cohort was measured using the Amplex Red Xanthine/Xanthine Oxidase Assay kit (Invitrogen A22182) according to the manufacturer’s instructions. Briefly, plasma samples (10 µl) taken from children upon hospital admission were incubated with hypoxanthine, the substrate of XO, Amplex Red reagent, and horseradish peroxidase (HRP). XO breaks down hypoxanthine into xanthine, which is then broken down into uric acid, and generates reactive oxygen species (ROS) along the way. In the presence of HRP, Amplex Red reacts with ROS to generate a fluorescent product (Excitation-530 nm/Emission-590 nm) that was quantified using a plate reader (Victor X3 from Perkin Elmer). ROS production was measured at different time points (1 h, 2 h, and for some samples also at 30 min) after the start of the reaction*.* ROS production in plasma from five healthy individuals were averaged and used as a reference to calculate relative units (RU) for each determination. RU at the different time points was averaged for each plasma sample and reported as the final XO activity. To determine XO levels at the one-month follow-up, we first selected the 100 patients with the highest levels of XO activity in plasma upon admission and determined XO levels from the 67 that had follow-up samples, all from children with severe malaria.

### Measurement of anti-PS and anti-DNA levels

Costar 3590 96-well ELISA plates were coated for16h at 4 °C with 100 µL of PS (Sigma) at 20 μg/ml in 200-proof Molecular Biology ethanol or calf thymus DNA (Sigma) at 10 μg/ml in PBS. DNA plates were allowed to evaporate at RT after coating. Plates were washed 5 times with 200 µL of PBS and then blocked for 1 h with 200 µL of PBS 3% BSA. Plasma from patients was diluted at 1:100 in PBS 1% BSA and incubated for 2 h at 37 °C. Plates were washed again five times and incubated with anti-human IgG-HRP (GE Healthcare) for 1 h at 37 °C. Plates were washed five more times and TMB substrate (BD Biosciences) was added until the desired color was obtained. The reaction was stopped by with Stop buffer (Biolegend) and absorbance was read at 450 nm. Positive and negative controls were the same used for XO determinations. The mean OD at 450 nm from triplicate wells was compared with the same dilution of the reference positive serum to calculate relative units (RU). Anti-PS and anti-DNA levels were determined in all available samples at one-month follow-up (n = 443).

### Measurement of IC levels

Costar 3590 96-well ELISA plates were coated with 100 µL of C1q capture antibody (1 µg/mL) (Thermofisher PA5-29586) in sodium bicarbonate-sodium carbonate buffer (pH 9.6, 50 mM, Bioworld 40520017-1) overnight at 4 °C. Plates were washed 2 times with PBS 0.05% Tween 20 washing buffer and incubated with 100 µL 0.1% BSA in PBS blocking buffer for 1.5 h at RT. Plasma samples were diluted 1:80 in 0.1% blocking buffer. 100µL were added to the wells and incubated for 2 h at RT. Plates were washed 2 times and incubated with 100uL polyclonal goat anti-human IgG-HRP diluted 1:5000 (Invitrogen) for 1 h at RT. Plates were washed 3 times before developing with TMB substrate as described for anti-PS. Positive and negative controls were the same used for XO determinations. The mean OD at 450 nm from triplicate wells was compared with the same dilution of the reference positive serum to calculate relative units (RU).

### Statistical analysis

Data were analyzed using Microsoft Excel, Graph Pad Prism and the StatsModels Python library. Differences in continuous variables between SMA group were analyzed using Wilcoxon rank sum analysis with Bonferonni correction to adjust for multiple testing. Differences in categorical variables were assessed using Fisher’s exact test or Pearson’s Chi squared test, as appropriate. Spearman’s rho was used to evaluate the correlation between the measured parameters and different clinical parameters. To evaluate differences in biomarker levels at admission and one-month follow-up, Wilcoxon signed rank test was used. To assess the interaction between XO levels and IC and anti-PS, we fitted two logistic regression models including the XO:IC and XO:anti-PS interaction terms, respectively. The p-value of the interaction term coefficient was considered as a measure of significant interaction. In addition, the log-likelihood of the fitted models was compared, correspondingly, to that of an additive model that did not include the interaction term. The log-likelihood ratio test was to evaluate model improvement upon inclusion of the interaction term.

## Results

**Figure 1:**
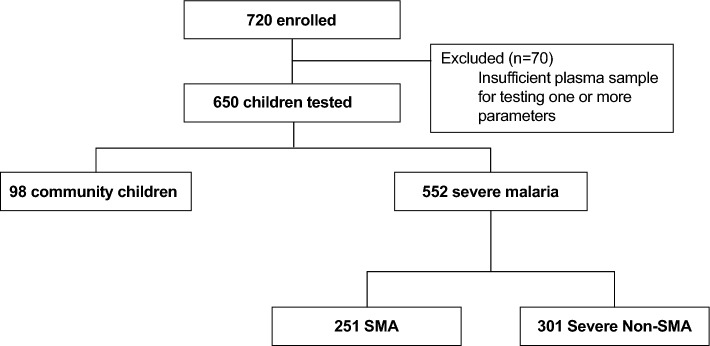
Flow chart of study participants.

In this study, we analyzed 650 plasma samples from Ugandan children under 4 years old. This cohort included 552 children hospitalized with a clinical diagnosis of severe malaria^24,28^, of which 251 presented with severe anemia (hemoglobin ≤ 5 g/dL), and 98 community children (Fig. 1). The present evaluation for SMA includes all children with severe malaria in the study across any category who had a hemoglobin of ≤ 5 g/dL, so could, for example, include some children with coma or multiple seizures. The children classified as “severe malaria, non-SMA”, had a hemoglobin of > 5 g/dL and one or more other complications of severe malaria. The community children were comparable in age and sex to the children with severe malaria (Table 1). In addition, children with severe anemia had lower parasite density but comparable parasite biomass to children without severe anemia.

**Table 1:** Summary of cohort demographics and laboratory parameters.

	CC (n = 98)	SMA (n = 251)	Non-SMA severe malaria (n = 301)	CC versus SMA *p* value	Non-SMA severe malaria versus SMA *p* value
Age (years)	2.19 (1.52, 3.10)	2.00 (1.35, 2.78)	2.06 (1.46, 2.94)	0.1528	0.3638
Female sex, No. (%)	41 (41.8)	105 (41.8)	139 (46.2)	0.9994	0.3059
Parasite density	0.00 (0.00, 0.00)	2483.00 (0.00, 60,736.00)	34,155 (877.00, 175,826.00)	< 0.0001*	< 0.0001*
Plasma HRP2 (ng/mL)	64.8 (64.8, 64.8)	1923.9 (363.9, 4847.6)	2593.0 (345.7, 6793.9)	< 0.0001*	0.1037
Creatinine (mg/mL)	0.26 (0.21, 0.32)	0.36 (0.27, 0.49)	0.38 (0.30, 0.48)	< 0.0001*	0.1984
BUN (mg/dL)	–	13.00 (7.00, 23.00)	11.00 (7.00, 19.00)	–	0.2581
Lactate (mmol/L)	–	6.50 (3.70, 12.00)	2.90 (1.80, 4.80)	–	< 0.0001*
Base excess (mmol/L)	–	-8.00 (-16.00, -4.00)	-8.00 (-11.00, -4.00)	–	0.2423
Bicarbonate (mmol/L)	–	15.90 (10.20, 18.90)	16.70 (13.70, 18.88)	–	0.0092
Hemolysis-related parameters					
RBC hemoglobin (g/dL)	10.9 (10.1, 11.6)	3.6 (2.8, 4.5)	8.4 (6.8, 10.2)	< 0.0001*	< 0.0001*
Haptoglobin (mg/mL)	476.2 (70.7, 1404.3)	4.0 (1.8, 13.7)	18.7 (5.0, 447.3)	< 0.0001*	< 0.0001*
Lactate dehydrogenase (U/L)	298 (266, 374)	823 (608, 1275)	548 (388, 838)	< 0.0001*	< 0.0001*
Plasma HO-1 (ng/mL)	6.7 (3.6, 9.3)	83.5 (41.9, 173.5)	61.2 (26.4, 120.0)	< 0.0001*	< 0.0001*
Total bilirubin (mg/dL)	0.2 (0.1, 0.3)	0.7 (0.3, 1.4)	0.6 (0.3, 1.1)	< 0.0001*	0.0847

Data are presented as median (1st quartile, 3rd quartile) for each demographic and laboratory characteristic except for percentage of female sex, which is presented as number of patients (percentage of the group). An asterisk (*) indicates significance (*p* ≤ 0.002), which was calculated using the Mann–Whitney test with Bonferroni correction (n = 24, adjusted α value = 0.002) for each continuous variable. For categorical variables, significance was assessed by chi-squared test. *CC* community controls, *SMA* severe malarial anemia, *BUN* blood urea nitrogen, *HO-1* heme oxygenase 1, *HRP2* histidine rich protein 2.

We conducted further analysis where we assessed clinical and laboratory indicators of hemolysis, and observed that children with SMA had lower RBC hemoglobin, lower levels of hemoglobin scavenger haptoglobin, and higher levels of lactate dehydrogenase (LDH), and heme-oxygenase 1 (HO-1), measures of RBC lysis and oxidative response, than community children or children with severe malaria but without SMA (Table 1).

### XO, anti-PS and IC are associated with SMA

A central goal of this study was to investigate whether the levels of XO are associated with severe anemia. We observed that children with SMA had significantly increased levels of XO compared to community controls and to children with severe malaria without SMA (Table 2). We also confirmed previous observations in other cohorts that both anti-PS antibodies and IC were associated with SMA^10,19–23^. However, we did not observe a significant association between anti-DNA antibodies and SMA and this parameter was excluded from further analysis related to SMA. When we performed these analysis using a more restrictive definition of SMA (which does not include children with *P. falciparum* densities lower than 10,000 parasites/µl; n = 96), we still observed increased levels of XO and anti-PS in this group compared to controls and children with severe malaria without SMA (Table S1).

**Table 2:** Comparison of measured parameters between CC, SMA, and non-SMA severe malaria patients.

	CC (n = 98)	SMA (n = 251)	Non-SMA severe malaria (n = 301)	CC versus SMA *p* value	Non-SMA severe malaria versus SMA *p* value
XO	0.72 (0.62, 0.82)	0.84 (0.71, 1.03)	0.78 (0.66, 0.93)	< 0.0001*	0.0014*
Anti-PS	0.37 (0.21, 0.52)	0.59 (0.38, 0.90)	0.53 (0.29, 0.78)	< 0.0001*	0.0151
Anti-DNA	0.42 (0.27, 0.55)	0.55 (0.40, 0.72)	0.51 (0.38, 0.69)	< 0.0001*	0.0779
IC	0.60 (0.48, 0.77)	0.73 (0.63, 0.84)	0.68 (0.53, 0.78)	< 0.0001*	< 0.0001*

Data are expressed in relative units (RU) and presented as median (1st quartile, 3rd quartile) for each parameter. An asterisk (*) indicates significance (*p* ≤ 0.006), which was calculated using the Mann–Whitney test with Bonferroni correction (n = 8, adjusted α value = 0.006). *CC* community controls, *SMA* severe malarial anemia, *PS* phosphatidylserine. All four parameters were determined in all samples from each group.

To further investigate the relation of the measured parameters with severe anemia, a logit model based on XO levels, anti-PS levels, and an XO:anti-PS interaction term showed that the latter did not significantly contribute to the SMA outcome (XO:anti-PS: *p* = 0.14; XO: *p* < 10^−3^; anti-PS: *p* = 0.009). Moreover, this model was not significantly better than an additive model where the interaction was not present (log-likelihoods ratio test: chi-squared = 2.25, *p* = 0.13). Similar results were observed with a model based on XO and IC (XO:IC: *p* = 0.076; XO: *p* = 0.001; IC: *p* < 10^−3^). Overall, these findings indicate that XO, anti-PS and IC levels are independently associated with severe anemia during malaria.

### Relationship between XO, anti-PS and IC

**Figure 2:**
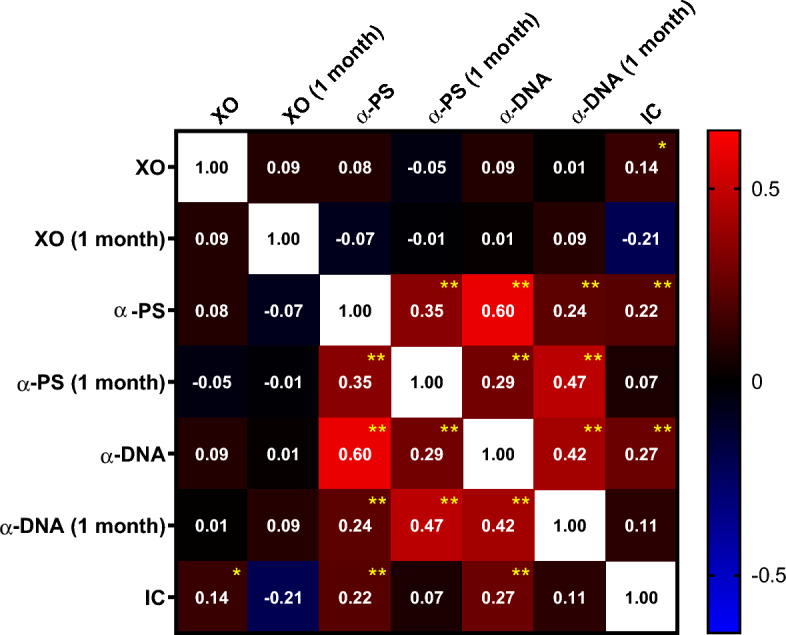
Correlation of XO, anti-PS, anti-DNA, and IC levels for all participants. Numbers indicate the Spearman’s ρ between the two indicated variables. Bonferroni-corrected α value is 0.001 (n = 49). The *p* values for each Spearman’s ρ are shown by yellow asterisks as follows: (*, *p* < 0.001; **, *p* < 0.0001).

We observed that the levels of XO are only weakly associated with the levels of anti-PS or IC (Fig. 2), suggesting that XO relation to severe anemia is independent of IC and autoimmune antibodies. Conversely, we observed a strong correlation between the levels of anti-PS and anti-DNA, either at the time of admission or 1 month after discharge, indicating that individual patients tend to have similar levels of the two autoantibodies and suggesting that some patients are more prone to autoreactivity.

### XO, anti-PS and IC correlate with clinical parameters associated with hemolysis

**Figure 3:**
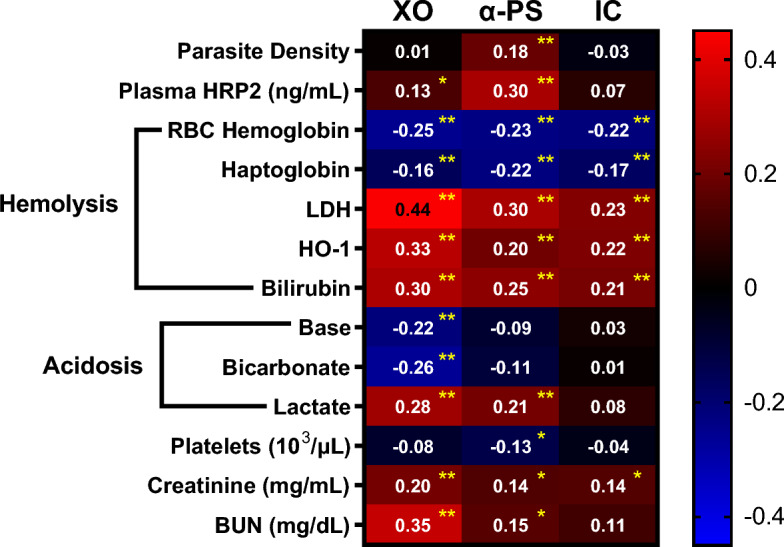
Correlation of levels of XO, anti-PS, and IC with RBC hemoglobin and parameters related to hemolysis, acidosis, and others. Numbers indicate the Spearman’s ρ between the two indicated variables. Bonferroni-corrected α value is 0.0013 (n = 39). The *p* values for each Spearman’s ρ are shown by yellow asterisks as follows: (*, *p* < 0.0013; **, *p* < 0.0001).

Correlation analysis between the levels of XO, anti-PS and IC, and clinical parameters related to anemia showed that all three are inversely correlated with RBCs hemoglobin, which is the clinical parameter that defines severe anemia (Fig. 3). They also correlate directly with three parameters that are associated with hemolysis: LDH, an enzyme that is released from lysed cells, including RBCs, and is often used as a marker of hemolysis^29^, HO-1, an enzyme that is upregulated in response to free heme^30^, and bilirubin, a product of heme degradation^31^. Haptoglobin binds to free hemoglobin in the circulation and is subsequently cleared. Therefore, decreased haptoglobin levels are associated with hemolysis^29,32^. An inverse correlation of XO, anti-PS and IC with haptoglobin was also observed (Fig. 3), indicating another association between each of the three parameters and hemolysis.

### XO correlates with clinical parameters associated with acidosis

We also analyzed the relation of XO, anti-PS and IC with parameters related with acidosis. Multiple factors appear to contribute to acidosis in malaria, both in the buildup and impaired clearance of excess lactate^33^. To counteract this acidification of the blood, the body uses circulating bicarbonate ions to neutralize lactic acid, but in doing so, generates more carbon dioxide. Consequently, patients with acidosis normally have high levels of circulating lactate and low levels of bicarbonate^33^.

We observed a significant correlation between XO and acidosis-related parameters (Fig. 3), suggesting a potential relation of this oxidative enzyme to acidosis. An association between anti-PS and lactate, but not base or bicarbonate, was found. No associations were found between acidosis parameters and IC.

### Relation to other clinical parameters and malaria complications

Comparing the levels of the measured parameters with clinical data revealed specific associations of each parameter. We observed that XO and anti-PS correlated significantly with parameters related with kidney function: blood urea nitrogen (BUN) and creatinine (Fig. 3), suggesting a possible relation of this oxidative enzyme with kidney damage. There were also a significant correlation between parasite biomass and anti-PS.

We also determined whether the measured parameters were related to specific malaria complications and clinical definitions of disease severity on admission (Table 3). In this table, if a child had the complication, they are included in the category, regardless of other complications they had. For example, most children with coma (cerebral malaria) also had multiple seizures. These children would be included in the coma and multiple seizures categories.

**Table 3:** Association of measured parameters with clinical complications in children with severe malaria.

	Patients with the complication (Yes)	Patients without the complication (No)	*p* Value
Acute kidney injury (n = 247/551)			
XO	0.83 (0.71, 1.11)	0.77 (0.67, 0.89)	< 0.0001*
Anti-PS	0.59 (0.36, 0.98)	0.54 (0.31, 0.76)	0.0197
IC	0.70 (0.57, 0.82)	0.70 (0.56, 0.80)	0.7429
Prostration (n = 395/552)			
XO	0.82 (0.71, 1.02)	0.72 (0.63, 0.85)	< 0.0001*
Anti-PS	0.57 (0.36, 0.89)	0.51 (0.23, 0.70)	0.0029
IC	0.71 (0.58, 0.82)	0.67 (0.55, 0.79)	0.0528
ACIDOSIS (n = 252/551)			
XO	0.82 (0.72, 1.12)	0.76 (0.67, 0.89)	< 0.0001*
Anti-PS	0.57 (0.34, 0.91)	0.54 (0.32, 0.78)	0.0710
IC	0.69 (0.57, 0.80)	0.71 (0.57, 0.80)	0.4106
Jaundice (n = 124/551)			
XO	0.88 (0.74, 1.21)	0.77 (0.68, 0.92)	< 0.0001*
Anti-PS	0.65 (0.40, 0.96)	0.54 (0.32, 0.79)	0.0345
IC	0.74 (0.66, 0.85)	0.69 (0.55, 0.79)	< 0.0001*
Coma (cerebral malaria) (n = 79/552)			
XO	0.81 (0.74, 1.09)	0.78 (0.68, 0.96)	0.0282
Anti-PS	0.65 (0.49, 0.96)	0.53 (0.31, 0.82)	0.0047
IC	0.73 (0.55, 0.80)	0.70 (0.57, 0.81)	0.6624
Multiple seizures (n = 232/552)			
XO	0.77 (0.67, 0.90)	0.83 (0.70, 1.01)	0.0026
Anti-PS	0.55 (0.29, 0.78)	0.56 (0.35, 0.86)	0.1462
IC	0.68 (0.54, 0.78)	0.71 (0.59, 0.83)	0.0043

After the name of each complication, the number of children with the complication versus the total number of children is indicated in parenthesis. Children could and often did have more than one complication. Data are expressed in relative units (RU) and presented as median (1st quartile, 3rd quartile) for each parameter. The fraction shown next to each complication represents the number of patients presenting with that particular manifestation of malaria over the total number of recorded Yes/No answers. An asterisk (*) indicates significance (*p* ≤ 0.0027), which was calculated using the Mann–Whitney test with Bonferroni correction (n = 18, adjusted α value = 0.0027).

We observed that XO levels were significantly higher in severe malaria patients with acute kidney injury, prostration, acidosis or jaundice, indicating a relation of this enzyme to these severe manifestations of malaria. However, XO levels were not associated with multiple seizures or with the most severe neurological complication, coma (cerebral malaria). IC were also significantly elevated in children with jaundice, indicating a relation of IC to this complication.

### Levels of XO and anti-PS after at 1-month follow-up

The levels of XO and anti-PS after 1 month showed a marked decrease compared to the samples collected at hospital admission (*p* < 0.0001 for both parameters), but still showed significant correlations with the levels at the time of admission (Fig. 2), indicating that levels of these parameters were still elevated for some patients. Median levels of XO in severe patients after 1 month were only slightly elevated when compared with community controls (Table 4), indicating that XO decreases almost to background levels in plasma after recovery. Levels of XO after 1 month still showed a significant difference compared to community controls (Table 4), but the difference was more significant for anti-PS, indicating persistence in plasma that is typical of these antibodies^23^.

**Table 4:** Comparison of levels of XO and α-PS antibodies in children with severe malaria at 1 month follow up to corresponding levels of these parameters in the CC group.

	CC	Severe malaria, 1 month follow-up	*p* Value
XO (n = 41)	0.72 (0.62, 0.82)	0.79 (0.66, 0.94)	0.0125*
Anti-PS (n = 189)	0.37 (0.21, 0.52)	0.47 (0.31, 0.65)	< 0.0001*

Data are expressed in relative units (RU) and presented as median (1st quartile, 3rd quartile) for each parameter. An asterisk (*) indicates significance (*p* ≤ 0.025) which was calculated using the Mann–Whitney test with Bonferroni correction (n = 2, adjusted α value = 0.025). *PS* phosphatidylserine.

## Discussion

Since the oxidative enzyme XO is frequently elevated in malaria patients and correlates with disease severity^18^, we hypothesized that XO could contribute to severe anemia in malaria patients. The results presented here indicate that the levels of XO in plasma are associated with SMA, but also with hemolysis-related parameters (haptoglobin, LDH, HO-1 and bilirubin), as well as with related complications such as jaundice.

Oxidative stress has been proposed as a contributor to SMA, since it causes peroxidation of lipids on the surface of erythrocytes, reducing the deformability of these cells^8^, and ultimately leading to their clearance in the spleen^7^. This hypothesis is supported by previous patient-based studies showing that, in the context of *P. falciparum* infections, RBCs rigidity was mostly observed in uninfected RBCs and correlated strongly with severe anemia^34^ and death^35^. Some potential factors that contribute to the oxidative burden during malaria include elevated levels of oxidative enzymes, free heme released after the lysis of infected and uninfected RBCs, and the phagocytic oxidative burst that is induced after phagocytosis of infected RBCs by innate immune cells^13^. It is still unclear what is the relative contribution of these factors to the high levels of oxidative stress that are observed in malaria patients, but XO is probably an important contributor, since this enzyme degrades hypoxanthine and xanthine in plasma, releasing high levels of reactive oxygen species. XO is elevated in the blood of malaria patients, correlating with disease severity^18^ and with inflammatory cytokines^36^. Our results further support that XO may be an important source of oxidative stress in malaria given its association with severe anemia, hemolysis and acidosis parameters.

Since the relation between XO and anemia is associative, it is possible that XO directly contributes to the loss of RBC, but also that elevated levels of XO are a consequence of anemia or other associated factors. A direct relation involving the release of XO by lysed RBC is not likely since XO activity is not found in RBC^17^. It is also unlikely that the observed increase in XO levels in patients may be caused by high levels of its substrates hypoxanthine/xanthine in plasma, since the concentration of hypoxanthine in uninfected RBCs that would be released upon lysis is very low (9.3 nM)^37^ and there is a marked decrease in the levels of hypoxanthine in plasma during *P. falciparum* infections^38^, probably caused by the robust transport of hypoxanthine and its precursors into infected RBC^39^. However, mice studies showed that the enzyme precursor of XO, xanthine dehydrogenase, is upregulated early during *Plasmodium* infection by the activation of type I IFN receptor^40^, proposing an immune-related mechanism for the upregulation of XO during malaria. Since hemolysis can induce secretion of type I IFN in liver macrophages^41^, it is also possible that the correlation between hemolysis and XO levels may be driven by the levels of this cytokine. However, if this was the case, a strong correlation between XO levels and other parameters associated with hemolysis (anti-PS and IC) would have been expected.

Oxidative stress has been proposed to induce SMA by causing increased rigidity of these cells that are subsequently recognized by phagocytes and cleared from the circulation^7,8^. It is possible that XO contributes to this mechanism, but in this study we did not quantify any parameter specifically associated to RBC phagocytosis in patients. However, we observed a significant association between XO levels and hemolysis parameters (haptoglobin, LDH, HO-1, bilirubin) that are compatible with the hypothesis that lysis of uninfected RBCs is directly induced by XO, as was observed in mice studies in the context of sickle cell disease^42^.

Other proposed causes of SMA include the binding of autoimmune antibodies to uninfected RBCs^1^, which increase their susceptibility to phagocytosis^9^ and their levels are associated with anemia in patients^43^. Mechanistic studies in a mouse model showed that binding of antibodies to the phospholipid PS, that is exposed in uninfected RBCs during malaria, increases RBCs phagocytosis and delays recovery from anemia^20^. In patients with *P. falciparum* and *P. vivax* infections, levels of anti-PS are associated with anemia^19–23^. However, we observed that they also correlate with parameters of hemolysis in patients, as described before^22^, suggesting they may contribute to SMA through both enhancing phagocytic clearance and hemolysis.

IC are also thought to contribute to phagocytosis of uninfected RBCs^11^ and correlate with SMA^10^. Here we observed that they correlate with parameters of hemolysis and also with anti-PS and anti-DNA, which probably indicates that part of the circulating IC are formed by these autoantibodies. It has been proposed that IC could contribute to anemia by increasing complement susceptibility of RBCs and increasing phagocytosis^44^. In this cohort, we also observed a correlation between IC and severe anemia, as well as hemolysis associated parameters.

The observed association of XO with different malaria complications points to a broader detrimental effect of this enzyme in severe malaria. It is possible that XO may contribute to complications such as jaundice and acute kidney injury as a consequence of increased hemolysis, since these complications are associated with it^45,46^. It is also possible that since XO is a highly oxidative and inflammatory enzyme^36^, the association with complications such as acute kidney injury may be driven by increased inflammation, which is thought to contribute to this complication^47^.

The novel finding of an association between XO and different severe malaria complications may open a possibility for adjunctive treatment, provided that a causative effect of XO in malarial anemia could be inequivocally demonstrated. Inexpensive and well-tolerated inhibitors of XO have been developed for the treatment of gout, since this condition is caused by accumulation of urate, the product of XO^48^. In mice studies, treatment with the XO inhibitor febuxostat reduced hemolysis induced in the context of sickle cell disease^42^. Given that elevated levels of XO may contribute to the development of severe anemia, treatment with the XO inhibitors allopurinol or febuxostat^49^, could contribute to alleviate this complication in malaria. Since febuxostat is not approved for use in children by the US Food and Drug Administration, and has a black box warning for adults, allopurinol is likely to be the preferred drug for studies in animals to evaluate prior to human use for malaria.

### Supplementary Information

Supplementary Information.

## Data Availability

Deidentified data are available from Dr. Chandy John on reasonable request.
