# Supplementary material for: Xanthine oxidase levels and immune dysregulation are independently associated with anemia in Plasmodium falciparum malaria
Source: Sci Rep. 2023 Sep 7;13:14720. doi: 10.1038/s41598-023-41764-4 (PMC10484935; doi:10.1038/s41598-023-41764-4)
Supplement: Supplementary file 1 — Supplementary Information. [file 41598_2023_41764_MOESM1_ESM.docx]

**Table S1.**

|  | **CC (n=98)** | **SMA (n=96)** | **Non-SMA**  **Severe**  **Malaria**  **(n=301)** | **CC vs. SMA P-value** | **Non-SMA**  **Severe**  **Malaria vs.**  **SMA P-value** |
| --- | --- | --- | --- | --- | --- |
| Xanthine Oxidase | 0.72 (0.62,  0.82) | 0.84 (0.72, 1.08 | 0.78 (0.66,  0.93) | <0.0001* | 0.0056* |
| α-PS antibodies | 0.37 (0.21,  0.52) | 0.65 (0.42, 0.92) | 0.53 (0.29,  0.78) | <0.0001* | 0.0042* |
| Anti-DNA | 0.42 (0.27,  0.55) | 0.56 (0.40, 0.76) | 0.51 (0.38,  0.69) | 0.0002* | 0.1316 |
| Immune complexes | 0.60 (0.48,  0.77) | 0.71 (0.61, 0.84) | 0.68 (0.53,  0.78) | 0.0008* | 0.0144 |

**Comparison of measured parameters between CC, SMA (using WHO definition), and non-SMA severe malaria patients.** In this analysis, the SMA group includes children that meet the WHO criterion for SMA (hemoglobin ≤5 g/dL and peripheral parasitemia by blood smear Giemsa staining >10,000 parasites/µL) (n = 96). Data are expressed in relative units (RU) and presented as median (1st quartile, 3rd quartile) for each parameter. An asterisk (*) indicates significance (p ≤ 0.006), which was calculated using the Mann-Whitney test with Bonferroni correction (n=8, adjusted α value=0.006). CC – Community controls, SMA – Severe malarial anemia, PS – phosphatidylserine. All four parameters were determined in all samples from each group.
